# Use of Safety Stand-Down Initiatives to Reduce Hospital Harm Events

**DOI:** 10.31486/toj.26.0029

**Published:** 2026

**Authors:** Armin Schubert, Leslie Norman, Diego Lara, Felecia Denson, Terrence M. Truxillo, Emily Bugeaud, Renee Digiovanni, Lori Minor, Slavvy Petrov, Deborah Ford

**Affiliations:** ^1^ Department of Anesthesiology, Ochsner Clinic Foundation, New Orleans, LA; ^2^ The University of Queensland Medical School, Ochsner Clinical School, New Orleans, LA; ^3^ Nursing Administration, Ochsner Clinic Foundation, New Orleans, LA; ^4^ Ochsner Children's Hospital, Ochsner Clinic Foundation, New Orleans, LA; ^5^ Quality and Performance Improvement, Ochsner Clinic Foundation, New Orleans, LA; ^6^ Multi-organ Transplant Institute, Ochsner Clinic Foundation, New Orleans, LA

**Keywords:** *Accidental falls*, *catheter-related infections*, *catheterization–central venous*, Clostridioides difficile, *clostridium infections*, *cross infection*, *delivery of health care*, *infection control*, *patient harm*, *patient safety*, *quality improvement*

## Abstract

**Background:**

Continuous improvement with short-cycle process change has become an accepted and commonly practiced quality tool in health care settings. While these improvement cycles had somewhat reduced our hospital's harm events over the years, patients continued to experience them. Staff and leaders were preoccupied with multiple competing priorities, especially in the aftermath of the COVID-19 pandemic. We report the use of safety stand-down initiatives to focus team attention and complement overall improvement efforts.

**Methods:**

The safety stand-down concept was adapted from industry to rapidly focus attention on safety and further reduce hospital harm events. We describe a stand-down process that harnesses stakeholder commitment, team attention, and resources so that stand-down actions can be initiated quickly. Four hospital-wide and 3 unit-based safety stand-downs were initiated in response to outliers or rapidly rising counts of hospital-acquired *Clostridioides difficile* infection, central line–associated bloodstream infection (CLABSI), and inpatient falls. Run charts were used to compare performance 12 months before and 6 to 9 months after the hospital-wide stand-down interventions.

**Results:**

Postintervention, *C difficile* infections, CLABSIs, and inpatient falls improved significantly based on meeting the run chart rules for constituting a shift. Reductions (39.2%, 78.9%, and 20.4%, respectively) were statistically significant at the *P*<0.05 level.

**Conclusion:**

Our experience demonstrates that safety stand-down initiatives can synergize with ongoing improvement cycles to mitigate adverse trends in hospital harm events.

## INTRODUCTION

Continuous improvement with short-cycle process change has become an accepted and commonly practiced quality tool in health care settings. While improvement cycles have reduced our hospital's harm occurrences over the years, patients continued to experience harm events.

### Local Problem Description

While many factors and process failures can lead to adverse trends in harm occurrence, we observed what appeared to be special-cause variation. We suspected this variation because run charts for *Clostridioides difficile* infections, central line–associated bloodstream infections (CLABSIs), inpatient falls, and falls with harm showed outliers, including some outside the 2 standard deviation control chart brackets. Furthermore, we assessed from event reporting and daily safety huddles that themes around substantive disruptions appeared to recur. Staff and leaders were preoccupied with multiple competing priorities, especially in the aftermath of the COVID-19 pandemic. US Centers for Disease Control and Prevention (CDC) data show that hospital-acquired infections increased in the United States during and after the pandemic.^1^ Temporary personnel, despite training and orientation, were unable to transfer their experiences into desirable process adherence. Supply shortages and substitutions, financial constraints, and training gaps hindered the application of best practices. To systematically determine which of the well-known root causes for our specific harm events of concern were operative, the hospital instituted a standardized, multidisciplinary, rapid assessment and action protocol at the launch of each initiative. Rather than relying on prolonged epidemiologic data collection, clinical leadership deployed real-time bedside practice audits, focused supply chain inventory reviews, and point-of-care staff interviews. This rapid assessment model allowed teams to differentiate between baseline process noncompliance and acute operational disruptions (such as postpandemic temporary personnel onboarding gaps or global product backorders), thereby establishing a clear baseline imperative for immediate intervention.

### Safety Stand-Down Rationale

To assist with course correction in response to rapidly deteriorating harm trends or outliers, we introduced the concept of a safety stand-down to our hospital unit teams and leadership. The safety stand-down concept was adapted from industry and the military to quickly focus attention on hospital harm events.^2^ The US Department of Labor defines a safety stand-down as a voluntary event during which employers communicate directly with employees about safety.^3^ An Occupational Safety and Health Administration communication explains that during temporary cessations of routine work, companies can have conversations with employees about safety, deliver training, conduct safety equipment inspections, develop rescue plans, or discuss job-specific hazards.^4^ A national safety stand-down initiative to reduce falls in the construction industry reached more than a million workers in the United States.^5^

Safety stand-downs have been used in the health care setting to improve adherence to hand hygiene^6^ and to enhance laboratory testing process safety.^7^ Safety stand-downs differ from rapid-cycle quality improvement in that stand-downs are used in response to impactful or outlier events or deviations in quality performance and deliberately seek to create attention and prioritize effort.^8^ Operationally, while standard quality improvement relies on continuous, incremental Plan-Do-Study-Act (PDSA) cycles managed by dedicated quality teams, a safety stand-down represents an acute, executive-authorized operational halt. A safety stand-down temporarily reprioritizes institutional resources, legally pausing nonurgent administrative projects to mandate that cross-functional stakeholders immediately assess, discuss, and mitigate a specific clinical risk.

Safety stand-downs can be conceptualized within established organizational, human factors, and implementation science frameworks that emphasize attention, prioritization, and shared sense-making in complex adaptive systems. Health care delivery environments are characterized by high cognitive load, competing priorities, and frequent interruptions, all of which may dilute the effectiveness of routine quality improvement activities. Safety stand-downs function as an intentional disruption of normal operational cadence, temporarily reallocating cognitive, relational, and organizational resources toward a clearly defined safety concern. From a human factors perspective, stand-downs increase the salience of safety signals by reducing background noise and aligning patient-facing staff, leaders, and support services around a shared mental model of risk. From an implementation science standpoint, safety stand-downs represent a focused facilitation strategy that accelerates adoption of known best practices by increasing leadership visibility, reducing competing work priorities, and enabling rapid removal of operational barriers. For these reasons, we chose to use targeted safety stand-downs to address performance gaps in hospital-acquired events that seemingly did not respond to secular improvement processes.

### Quality Improvement Study Aim

The specific aim of this quality improvement study was to evaluate the 6- to 9-month impact of organization-wide safety stand-down events on the occurrence of hospital-associated harm events, specifically for *C difficile* infections, CLABSIs, and inpatient falls.

## METHODS

This quality improvement initiative was designed as a longitudinal observational study of harm event occurrences. We selected the longitudinal observational design because our safety stand-down interventions were implemented as operational patient safety responses rather than research-driven experimental interventions. Randomization or controlled interruption was neither feasible nor ethically appropriate given our intent to promptly mitigate patient harm. This design allows for evaluation of temporal associations between safety stand-down initiation and subsequent changes in harm event occurrence while preserving the real-world context in which these interventions were deployed. During the stand-down action periods, ongoing quality and safety improvement activities continued, such as daily safety huddles, event reporting, root cause analysis and focused review, and unit-based improvement projects using the Institute for Healthcare Improvement performance improvement format.

Institutional review board approval was not necessary as the interventions were conducted as part of the hospital's routine quality and patient safety operations. This initiative was reviewed and determined to constitute a quality improvement project rather than human subjects research. Interventions were implemented to improve standard care processes and involved no randomization or deviation from accepted clinical practices. The primary ethical imperative underlying the interventions was the timely reduction of preventable patient harm.

### Setting

The interventions and results reported are in the setting of a 550-bed tertiary/quaternary academic medical center that serves as a destination for referral throughout Louisiana and neighboring states. The organization is part of a major regional health system with the mission to serve, heal, lead, educate, and innovate and to influence innovation through a network of diversified stakeholders.

### Safety Stand-Down for Hospital-Acquired *Clostridioides difficile* Infection Events

#### Rationale for Intervention.

**Figure 1.**
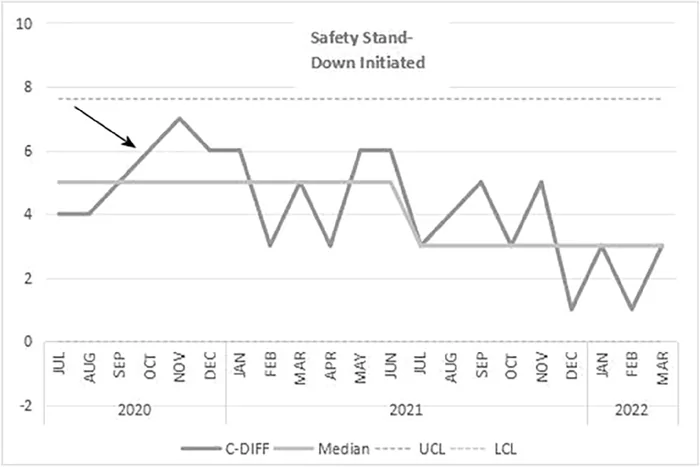
**Hospital-acquired *Clostridioides difficile* (C-DIFF) harm events before and after the safety stand-down initiative. The arrow points to event numbers outside the control limits. The events constituting a shift per run chart rules (below the baseline median) occurred in July, August, and October 2021 and in December, January, February, and March 2022.** LCL, lower control limit; UCL, upper control limit.

Hospital-acquired *C difficile* infections began to rise in mid-2020 (Figure 1). This increase was thought to be a consequence of disruptions in normal care processes during multiple COVID-19 pandemic spikes in the New Orleans, Louisiana, area. When another increase occurred a few months later in spring 2021 during a period of relative COVID-19 control, hospital quality leadership decided that a rapid intervention was needed to achieve greater safety for our patients.

#### Intervention.

After reviewing safety stand-down processes in industry and becoming familiar with the prior use of safety stand-downs in health care, we planned a hospital-wide patient safety stand-down. The planning process was accomplished rapidly; included a small group of nursing, infection prevention, performance improvement, and physician leaders; and was supported by the health system quality leadership. The stand-down process consisted of (1) executive sponsors publicly announcing and committing to a safety stand-down process even if other projects needed to be postponed, (2) initiating a kickoff meeting with relevant stakeholder leaders, (3) using a rapid-cycle improvement tool, and (4) convening stakeholder groups during a 6- to 9-week stand-down period to identify and implement stand-down actions.

**Figure 2.**
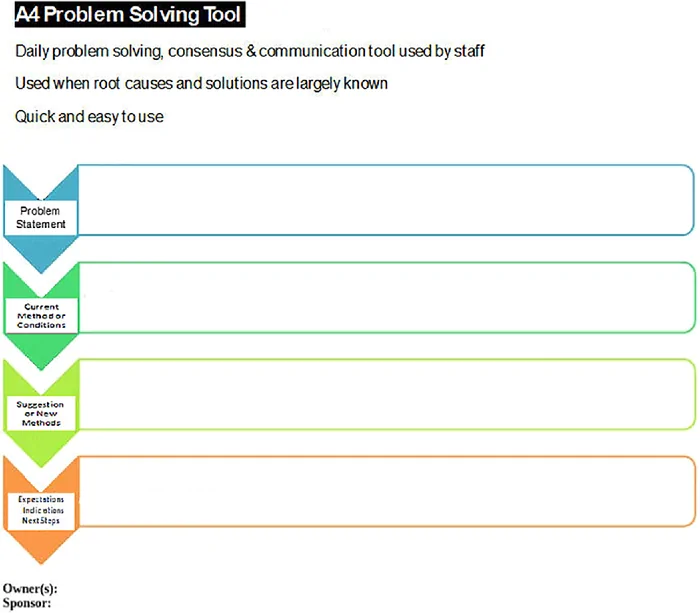
Lean A4 rapid-cycle improvement tool used in the stand-down process.

To provide operational structure, the stand-down sessions were executed via a highly structured communication and assessment framework:

1. Initial kickoff and focused dialog: The initial kickoff meeting convened relevant stakeholders, generally leaders of care groups that were likely to impact the known root causes for the 3 harm event types, including frontline nurses, medical directors, pharmacists, supply chain managers, and infection preventionists. Known data were shared from deep-dive case reviews and evidence-based safety bundles related to specific safety events. For example, for the *C difficile* infection stand-down, data on past *C difficile* infection events, hand hygiene, timing of stool cultures, and *C difficile* testing were shared by the infection prevention department. Process owners were assigned for each stakeholder group. Discussions focused on reviewing the acute data trends, mapping immediate care vulnerabilities, and establishing psychological safety so frontline staff could openly report workflow barriers without fear of punitive action.
2. Assessment and recommendation process: Teams used the Lean A4 format (Figure 2) to visually chart the known problem, current state, and target state. After the kickoff, stakeholders identified opportunities in their work environments and shared their findings at the next stand-down meeting, often as early as within 5 to 7 days. An example is an assessment by staff in the rehabilitation therapy department that indicated the possible spread of organisms from physical therapy equipment. This assessment led to the immediate purchase of dedicated equipment for *C difficile* patient rooms and an in-depth review of the process to disinfect equipment upon patient discharge. At the next meeting, stakeholders proposed and committed to stand-down actions they could implement quickly—within the defined stand-down period—with a reasonable stretch effort and that were likely to lead to harm avoidance. Actions were largely based on reviewed audit data collected during the preceding days or weeks. If an assessment revealed a systemic gap, such as a product shortage, the group formulated an actionable recommendation. Performance improvement and infection control sponsors provided guidance to stakeholder groups, typically during the earliest stand-down meetings.
3. Implementation mechanisms: Approved recommendations were translated into immediate operational changes. Rather than waiting for formal policy revisions, changes were implemented through just-in-time peer education, daily huddle briefs, and mandatory integration of bedside checklists. Stakeholder owners were assigned to each task to guarantee accountability and resource allocation within the compressed stand-down time frame. Executive sponsors and process owners focused on these commitments at follow-up meetings, reviewed process data, and removed barriers.
4. Final report and connection to standard quality improvement processes: A safety stand-down summary report was presented to hospital unit leadership at the end of the stand-down period. This report identified the stand-down actions implemented and the planned follow-on actions that could not be implemented within the stand-down time frame. Actions requiring longer time frames were referred to the hospital or health system quality groups for follow up outside of the stand-down framework.

We chose the Lean A4 format instead of the A3 format because the root causes for hospital harm events are relatively well known, meaning that the hospital quality improvement team had compiled and calibrated against evidence from the literature the root causes that accounted for almost all harm events of a specific type (eg, hospital-associated *C difficile* infection). This evidence-based method is frequently used in industry and health care, including UCLA Health, Cleveland Clinic, ThedaCare, Virginia Mason Medical Center, and Mayo Clinic. The purpose of using the Lean A4 format is to encourage and generate ideas from patient-facing staff to improve work, solve problems, reduce unnecessary interventions, and place value on teamwork and people.

The rationale identification and intervention processes used for the *C difficile* infection safety stand-down were also used for the other safety stand-downs.

### Safety Stand-Down for Hospital-Acquired CLABSI Events

**Figure 3.**
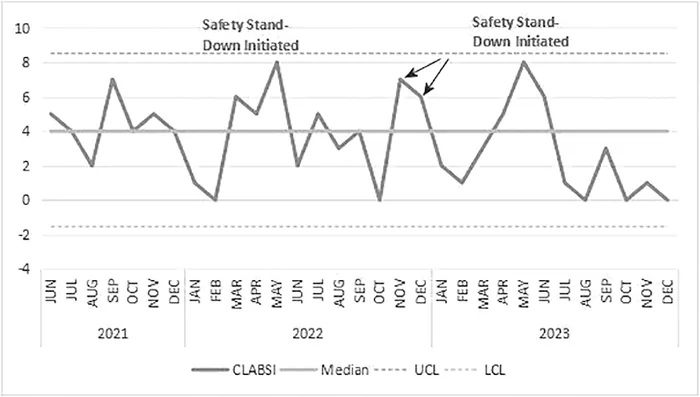
**Central line–associated bloodstream infection (CLABSI) harm events before and after safety stand-down initiatives in 2022 and 2023. The arrows denote the 2 months with a high number of events that led to the initiation of the second stand-down.** LCL, lower control limit; UCL, upper control limit.

Review of annual and monthly CLABSI event data showed a rate higher than our hospital peers, several astronomical values (ie, outlier events above the upper control limit),^9^ and, in the second quarter of 2022, a concerning number of events that appeared to be increasing rapidly (Figure 3). Therefore, a hospital-wide CLABSI safety stand-down was announced.

### Safety Stand-Down for Inpatient Falls

**Figure 4.**
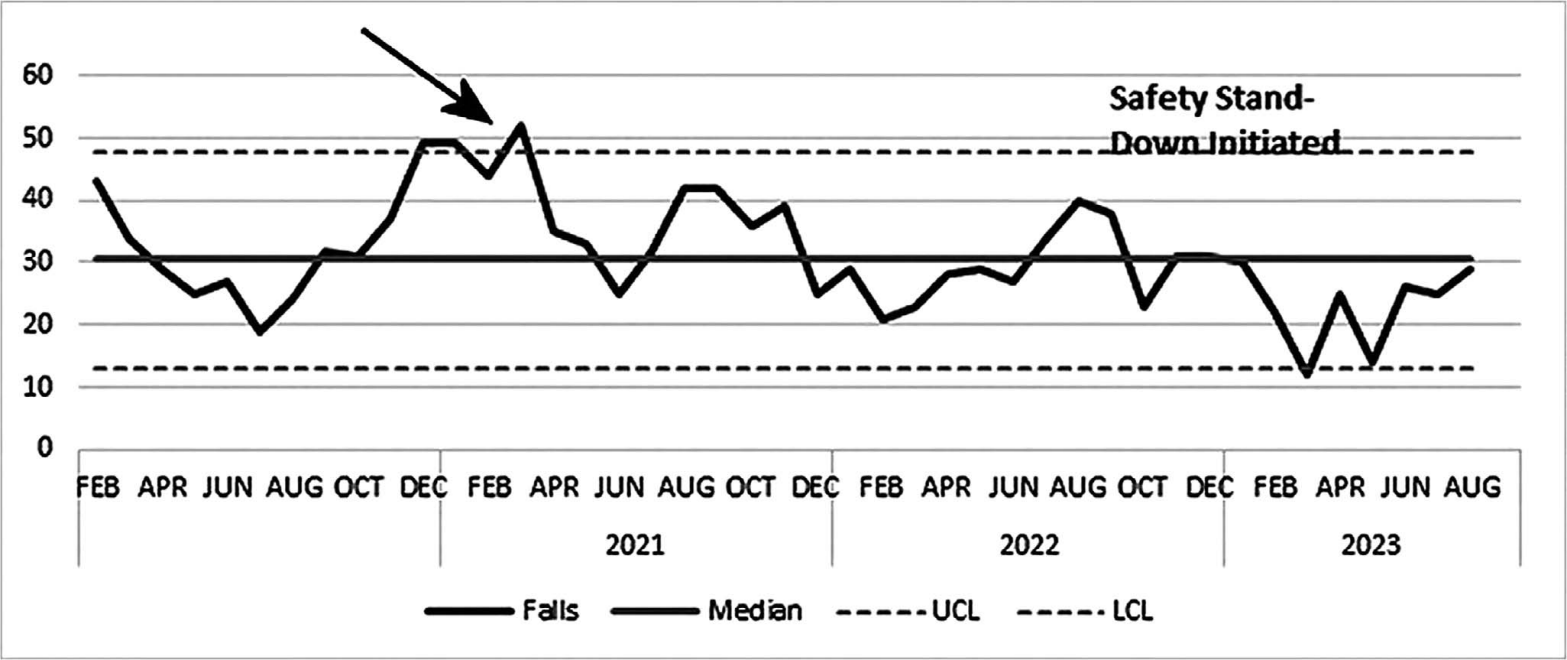
**Inpatient falls before and after the safety stand-down initiative. The arrow points to event numbers outside the control limits.** LCL, lower control limit; UCL, upper control limit.

**Figure 5.**
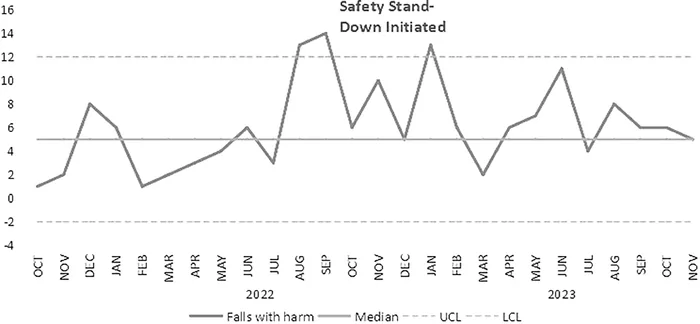
**Inpatient falls with harm before and after the safety stand-down initiative. Event numbers outside the control limits occurred in several months.** LCL, lower control limit; UCL, upper control limit.

While the number of annual falls in the hospital had been decreasing by an average of 10% to 15% per year since 2018, an astronomical value had been observed (Figure 4). At the same time, falls with harm were increasing substantially, showing astronomical values in August, September, and the following January (Figure 5). These signals prompted us to initiate a hospital-wide falls safety stand-down.

### Safety Stand-Downs for Specific Inpatient Units

While *C difficile* infection, CLABSI, and inpatient falls stand-downs were hospital-wide initiatives, 5 units in the hospital also adopted the safety stand-down method: an oncology unit, a medical stepdown unit, and 3 inpatient pediatric units. The 3 inpatient pediatric units accounted for the plurality (16/47) of hospital-acquired CLABSIs in 2022. During the first 7 months of 2023, an additional 5 CLABSI events occurred on the pediatric units. During stand-down assessments, this increase was attributed to several factors. At the time, Prevantics antiseptic skin swabs (Professional Disposables International, Inc), an important part of central line dressing care in our units, were backordered worldwide. The use of peripherally inserted central catheters (PICCs) in neonatal congenital heart patients increased; these 2.6 French catheters are very flexible and tend to telescope forward during dressing changes, regardless of how carefully they are handled, resulting in many CLABSI events from skin flora. Additionally, after auditing the Children's Hospitals’ Solutions for Patient Safety central line nursing bundle compliance, we found substantial room for improvement. These observations galvanized pediatric unit and quality leaders to adopt the safety stand-down process.

### Study Population

For the hospital-wide stand-down interventions, data from all inpatients in our hospital were included in this report for the time frames reported for each intervention: 9 to 12 months for baseline and 6 to 9 months for postintervention analyses. For the pediatric unit–based stand-down, we used a preintervention period of 9 months and a postintervention period of 12 months; data for all patients from the 3 pediatric units were included, and the data from the 3 units were aggregated.

### Safety Stand-Down Process Measures

We considered process measures for safety stand-down interventions to be the (1) ability to complete the stand-down event within 8 to 10 weeks, (2) participation of all identified stakeholders, and (3) completion of at least one stand-down action by each stakeholder group. We intentionally used stand-down interventions sparingly to address only truly concerning harm trends so as not to desensitize teams. Therefore, all stand-down interventions were temporally separated. To track the time series of harm events with an intervention occurring during a relatively short time interval (generally <9 weeks), we used run charts because data for baseline periods of adequate duration were available. Use of run charts also allowed visual inspection, including the identification of outlier events. While the hope is that interventions are sustainable, we viewed stand-down interventions as supportive of existing improvement processes and, because of their focused nature, more effective in the near term. These considerations prompted the selection of a 6- to 9-month postintervention analysis period for the hospital-wide events.

### Outcome Measures and Rationale

Our hospital's performance improvement and infection prevention departments track all harm events in real time and report results weekly to unit, medical staff, and hospital leaders. Harm event data are stored in a secure, central data repository. We defined falls with harm as falls that occurred in the inpatient hospital setting and met the criteria set by the National Database of Nursing Quality Indicators.^10^ We used the CDC National Healthcare Safety Network definitions of hospital-acquired CLABSI and *C difficile* infection events.^11,12^

When sharing data with our clinical teams and for the purpose of illustration in this report, we chose occurrence of events vs standardized incidence rates. Implementing high reliability organization principles is associated with reductions in harm events.^13^ High reliability organizations emphasize real-time recognition and review of individual harm events as part of the commitment to a zero-harm goal.^14^ Reporting harm events in near-real time allows more effective immediate feedback and learning for clinical teams^15^ because rate metrics such as standardized infection ratios are generally available only many months after harm events occur. Occurrence rates can also be perceived as abstract constructs unrelated to the experience of a patient as an individual human being. Moreover, a safety culture and better staff engagement have been associated with improved patient safety outcomes.^16^ Our leaders and frontline staff therefore focused on harm occurrences rather than incidence rates to make patient harm more personally relatable, visible, and actionable.

### Harm Event Data Analysis

Harm event data were primarily visualized in run charts to allow for data representation before and after the intervention. Median values were computed for a period of 12 months before the stand-down intervention (except for pediatrics that had only 9 months of baseline data available) and 6 to 9 months after the intervention (except for pediatrics that had a 12-month postintervention period). To better indicate variation and outliers, upper and lower control limits were calculated using 2 standard deviations from the mean and added to the run chart figures. Statistical run chart rules, including trend, shift, and number of runs, were used to evaluate the likelihood that the observed change was associated with the intervention, which was treated as a special-cause variation.^9^ We inferred statistical significance when a run chart rule was met. In addition, we calculated the percentage change in event occurrence using the monthly preintervention and postintervention event rate. Before and after rate differences were evaluated for statistical significance with the Fisher exact test adapted for Poisson rates.^17^

## RESULTS

From 2021 to 2023, Ochsner Medical Center-New Orleans used the safety stand-down method 7 times. Four hospital-wide safety stand-downs were conducted for hospital-acquired *C difficile* infection, CLABSI, and inpatient falls. In addition, specific inpatient unit safety stand-downs were conducted for *C difficile* infection (oncology unit), CLABSI (3 pediatric units), and inpatient falls with harm (medical stepdown unit).

Complete harm event data sets were available for all hospital-wide safety stand-downs and the pediatric CLABSI stand-down initiative (combined data from the 3 inpatient pediatric units). Leaders of the oncology and medical stepdown units that conducted stand-downs reported a substantial decrease in harm events (*C difficile* infections and falls with harm) after their units’ stand-down events. The unit stand-downs occurred at a different time than the hospital-wide stand-downs.

Table 1 lists process measures of success for the stand-down interventions with complete harm event data. Only 2 of 35 stakeholder groups (Information Services and Supply Chain) involved in the 5 reported stand-downs were unable to complete an action during the stand-down period.

**Table 1.** Safety Stand-Down Process Measures

Stand-Down	Completed	Number of Stakeholder Groups	Stand-Down Actions per Stakeholder Group	Time to Stand-Down Final Report, weeks
*Clostridioides difficile* infection	Yes	8	1-4	6
CLABSI, 2022	Yes	6	1-2	9
CLABSI, 2023	Yes	9	1-3	11
Inpatient falls	Yes	6	0-2	9
Pediatric CLABSI	Yes	6	0-3	6

CLABSI, central line–associated bloodstream infection.

### Hospital-Wide *Clostridioides difficile* Infection Prevention Safety Stand-Down, 2021

The *C difficile* infection safety stand-down was completed within 6 weeks from announcement to final report. Table 2 lists the stakeholder groups with their short-term (stand-down) and follow-on actions. The rapid assessment revealed that process disruptions and inconsistent Environmental Services cleaning during COVID-19 spikes contributed to rising infection rates. Examples of rapid actions included nursing stakeholders implementing a rigorous *C difficile* verification checklist, while Environmental Services standardized ultraviolet disinfection and adenosine triphosphate surface testing to validate cleanliness. This direct operational modification correlated with immediate clinical results. For 9 months after the hospital-wide 2021 *C difficile* infection safety stand-down, the number of events was below or at the baseline median 7 times. According to run chart rules for constituting a shift, values on the median line do not contribute to the shift, nor do they interrupt it. Therefore, a statistically significant shift was observed (Figure 1). *C difficile* events declined by 39.2% in the 9 months following the intervention: from 61 events during the 12-month baseline period to 28 events during the 9-month postintervention period (*P*=0.0298).

**Table 2.** Hospital-Wide *Clostridioides difficile* Infection Prevention Stakeholder Groups and Stand-Down Actions

Stakeholder Group	Stand-Down Action	Follow-On Action
Nursing	• Implement a *C difficile* verification checklist • Update standard operating procedure for *C difficile* patient management • Update diarrhea decision tree algorithm and guidance for appropriate testing • Revise test ordering escalation process	• Provide weekly adherence reports to nursing (reuse of a testing algorithm verification form) and share with medical staff unit leaders to improve testing stewardship
Medical staff	• Provide *C difficile* test ordering stewardship education to inpatient services	• Provide additional coaching to colleagues as needed (provided by medical staff unit leaders)
Laboratory Services	• Audit for appropriate stool samples and cancel tests if inappropriate	• Cancel tests for known positive patients
Environmental Services	• Approve appropriate cleaning and disinfection products • Standardize use of ultraviolet disinfection and adenosine triphosphate qualitative surface cleaning testing	• Monitor and report weekly utilization of touchless disinfection in isolation patient rooms
Infection Prevention	• Conduct isolation rounds every other day in units with *C difficile* patients • Redistribute resources to nursing units to help guide appropriate testing for *C difficile* • Evaluate isolation caddy inventory and work with Central Supply/Cleaning and Distribution to obtain alternative options if needed	• Evaluate/select sporicidal disinfectant product to be used across all units for daily, discharge, and equipment cleaning • Monitor and report weekly adherence to testing algorithm
Pharmacy	• Increase focus on inappropriate antibiotic prescribing, de-escalation, and duration • Pilot a gastric acid suppression stewardship service to identify opportunities to discontinue proton pump inhibitors and H2 antagonists without indications	• Continue increased focus on inappropriate antibiotic prescribing, de-escalation, and duration
Information Services	• Build a unit-based *C difficile* electronic medical record report	• Revise decision support system
Rehabilitation Therapy	• Keep rehabilitation equipment in *C difficile* patient rooms	• Revise ambulation protocols outside rooms

H2, histamine type 2.

**Figure 6.**
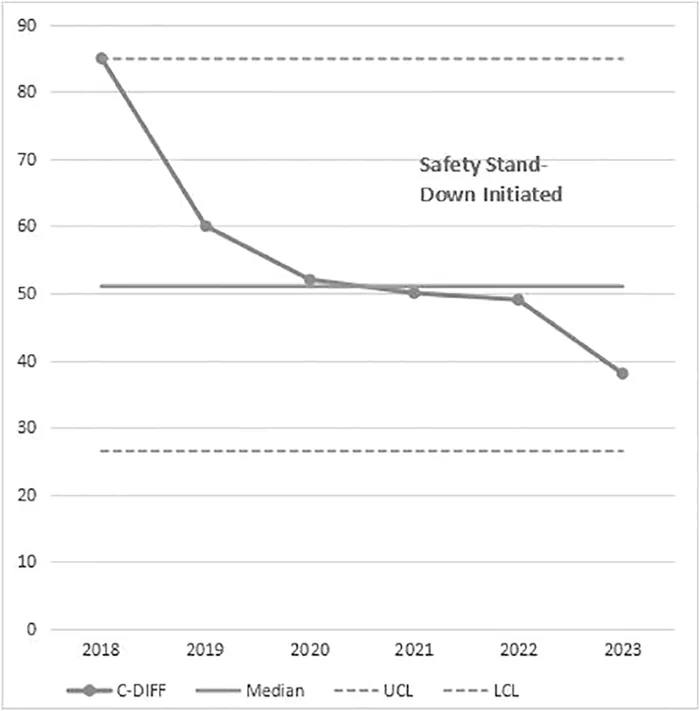
**Hospital-acquired *Clostridioides difficile* (C-DIFF) harm events, 2018 to 2023.** LCL, lower control limit; UCL, upper control limit.

The annual number of hospital-acquired *C difficile* infections continued to decline for 2 years after the stand-down event (Figure 6). This acute, short-term reduction effectively reset the operational baseline, initiating a downward trajectory that successfully translated into a sustained multiyear reduction in aggregate annual cases, as visualized in the long-term trend data.

### Hospital-Wide CLABSI Prevention Safety Stand-Downs, 2022 and 2023

**Figure 7.**
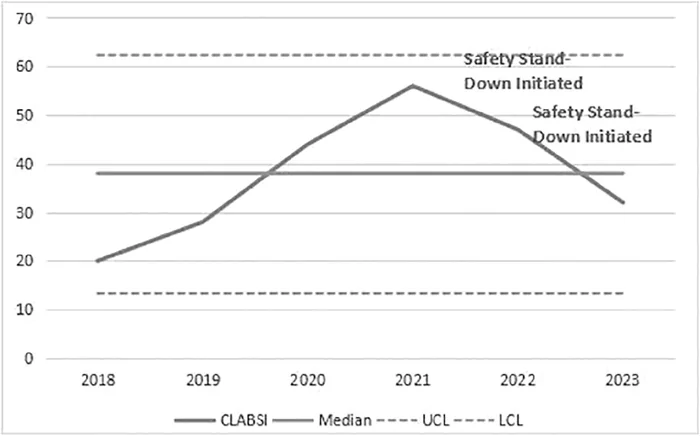
**Hospital-acquired central line–associated bloodstream infection (CLABSI) harm events, 2018 to 2023.** LCL, lower control limit; UCL, upper control limit.

The CLABSI safety stand-downs in 2022 and 2023 were accomplished within 9 weeks and 11 weeks, respectively, from announcement to final report. The stakeholder groups are listed in Tables 3 and 4, along with their short-term (stand-down) and follow-on actions. The first CLABSI stand-down in 2022 was followed by a 5-month period during which monthly CLABSI events appeared to decrease from the high number that prompted the stand-down. However, using statistical run chart rules, no significant change could be detected. After 2 months with a high number of CLABSI events, another hospital-wide safety stand-down was initiated in 2023 that involved more stakeholders. CLABSI events decreased from a median of 4 to 1, a level substantially below the baseline median and constituting a shift by run chart rules (Figure 3). CLABSI events declined by 78.9% in the 6 months following the second safety stand-down: from 46 events during the 12-month baseline period to 5 events during the 6-month postintervention period (*P*=0.0004). The number of events during the 12 months prior to the first intervention was 47, highlighting the fact that the first intervention had little or no impact. The 5-year CLABSI harm trend continued downward (Figure 7), showing a reduction of approximately 45%: from 56 events in 2021 to 31 events in 2023.

**Table 3.** Hospital-Wide Central Line–Associated Bloodstream Infection Prevention Stakeholder Groups and Stand-Down Actions, 2022

Stakeholder Group	Stand-Down Action	Follow-on Action
Nursing	• Commit to daily chlorhexidine gluconate baths for all patients	• Revamp nursing education for blood culture draws
Medical staff	• Restrict blood culture ordering to primary team • Develop central line removal protocol	• Increase awareness among medical staff to search for alternative sources for bacteremia with Epic (Epic Systems Corp) report
Infection Prevention	• Increase central line audits by 50% on priority floors	• Provide real time audit results to nursing staff and unit leaders for follow up
PICC team	• Begin weekly dressing changes for all patients with central lines	• Complete PICC team hiring
Information Services	• Build a report of patients with central lines who had recent blood cultures ordered	• Automate notification to providers
Supply Chain	• Identify better central line dressings for larger catheters	• Investigate supplier options

PICC, peripherally inserted central catheter.

**Table 4.** Hospital-Wide Central Line–Associated Bloodstream Infection (CLABSI) Prevention Stakeholder Groups and Stand-Down Actions, 2023

Stakeholder Group	Stand-Down Action	Follow-on Action
Nursing	• Create blood culture contamination huddle helper • Provide in-service retraining in all critical care areas	• Expand nursing skills fairs
Medical staff	• Evaluate patients with central lines on admission for blood culture screening • Include CLABSI risk in daily rounds • Consider alternative site selection (especially subclavian)	• Adopt Michigan Appropriateness Guide for Intravenous Catheters • Achieve greater real-time coordination of infection preventionist review with medical staff to enhance search for alternative sources • Receive central line feedback from infection preventionist
Infection Prevention	• Reinforce aseptic central line access practices	• Round with a rolling cart to assess competency and address questions about scrubbing the hub
PICC team	• Perform line surveillance and dressing changes daily Monday through Friday • Provide just-in-time education for nurses	• Adopt PICC huddles for appropriate units • Provide clinical decision support for line selection
Pharmacy	• Reinforce best practices for compound sterile products • Support antimicrobial stewardship practices	• Work with providers to bundle medication orders (reduced line access)
Laboratory Services	• Increase blood culture contamination reporting frequency to weekly for prioritized units	• Develop cost reports for blood culture contamination and share with each unit
Environmental Services	• Prioritize cleaning completion audits for prioritized units	• Expand audits to all critical care areas
Information Services	• Build Epic (Epic Systems Corp) central line best practice advisory for nursing care plan • Modify admission orders for patients with central lines on admission	• Flag line on interunit transfer • Flag emergently placed lines
Supply Chain	• Stock Kurin needles (Kurin, Inc) in ICUs • Increase PAR level stocking of Curos caps (3M Company)	• Provide education for new central line dressing change kit • Leverage vendor partnerships

ICU, intensive care unit; PAR, periodic automatic replacement; PICC, peripherally inserted central catheter.

### Hospital-Wide Inpatient Falls Prevention Safety Stand-Down

**Figure 8.**
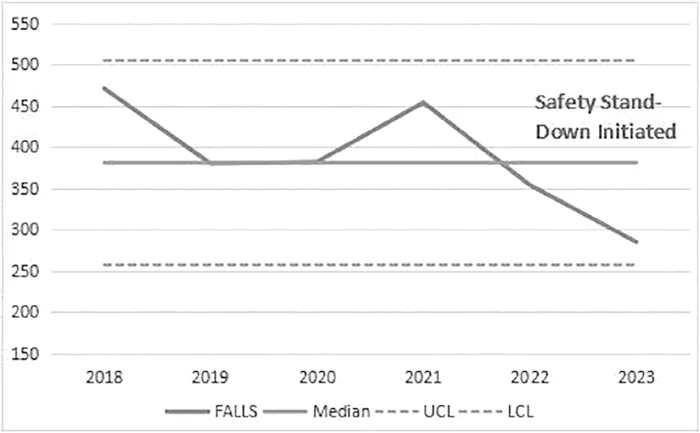
**Inpatient falls, 2018 to 2023.** LCL, lower control limit; UCL, upper control limit.

**Figure 9.**
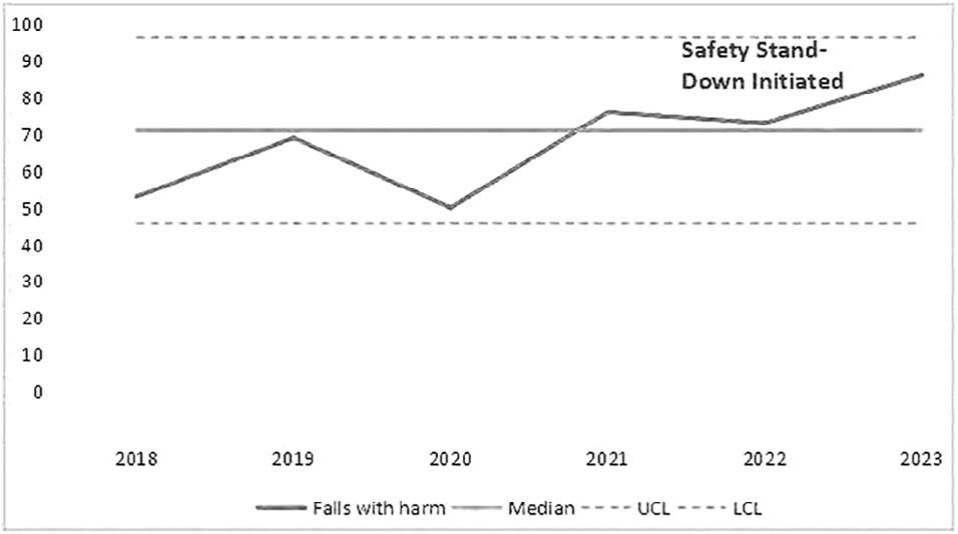
**Inpatient falls with harm, 2018 to 2023.** LCL, lower control limit; UCL, upper control limit.

The inpatient falls prevention safety stand-down was completed in 9 weeks from announcement to final report. The stakeholder groups are listed in Table 5, along with their short-term (stand-down) and follow-on actions. Initial assessment indicated that hospital-wide postpandemic staffing instability heavily impacted patient surveillance. To address this problem, nursing and remote monitoring stakeholders codesigned an intervention to increase remote video sitter utilization to 100% among high-risk cohorts, and the pharmacy staff conducted systematic polypharmacy reviews. This highly focused, attention-driven strategy yielded immediate success. Beginning in January 2023, the number of monthly inpatient falls declined below the baseline median in 7 monthly observations, constituting a shift according to run chart rules (Figure 4). Inpatient falls declined by 20.4% in the 9 months following the intervention: from 371 events during the 12-month baseline period to 221 events during the 9-month postintervention period (*P*=0.0066). Annual inpatient falls decreased from 354 in 2022 to 275 in 2023 (Figure 8). Inpatient falls with harm did not decrease after the safety stand-down (Figure 5), although outlier conditions did not occur after February 2023. The data show an increasing 5-year trend for inpatient falls with harm (Figure 9).

**Table 5.** Hospital-Wide Inpatient Falls Prevention Stakeholder Groups and Stand-Down Actions

Stakeholder Group	Stand-Down Action	Follow-On Action
Nursing	• Cohort patients with high fall risk (including fall history) • Pilot patient and family contracts regarding fall safety	• Increase remote sitter monitoring utilization to 100%
Medical staff	• Ensure mobility orders are placed for all patients • Obtain toxicology consults for patients with delirium contributing to falls	• Audit for mobility orders on executive rounds • Incorporate toxicology consult as a checkbox in falls review
Remote monitoring team	• Reeducate monitoring techs	• Staff up remote monitoring tech team
Pharmacy	• Review patient medication regimens for polypharmacy	• Incorporate pharmacy as a checkbox in falls review
Information Services		• Develop assistive technology to increase effectiveness of monitoring techs
Supply Chain	• Ensure adequate PAR levels of assistive equipment	NA

NA, not applicable; PAR, periodic automatic replacement.

### Pediatric Unit CLABSI Prevention Safety Stand-Down

**Figure 10.**
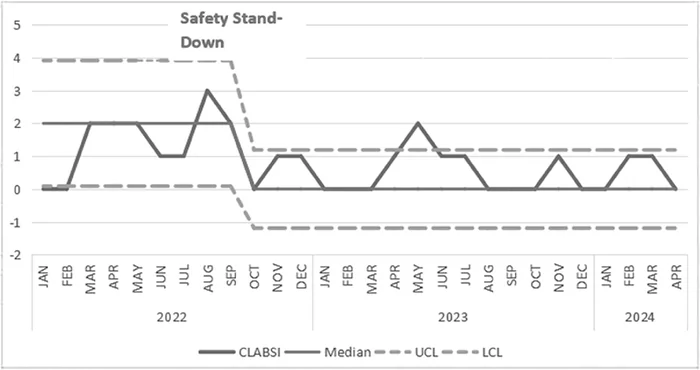
**Pediatric central line–associated bloodstream infection (CLABSI) harm events before and after the safety stand-down initiative.** LCL, lower control limit; UCL, upper control limit.

The pediatric CLABSI prevention safety stand-down was conducted in 3 pediatric units (pediatric intensive care, pediatric cardiovascular intensive care, and pediatric acute care) and was completed in 6 weeks. Data from all 3 units were aggregated. Stakeholders and stand-down actions are listed in Table 6. Initial efforts were directed to improve bundle compliance by creating Kamishibai (K) cards for team members performing central line care; these cards were used to guide discussions during rounds on each patient with a central line. K cards are used in manufacturing environments as a management tool; in health care they can be used to ensure bundle elements have been addressed during daily rounding.^18^ Audits were conducted to help ensure compliance. We joined a multicenter quality improvement project along with our partner hospitals in the southeast region of the Children's Hospitals’ Solutions for Patient Safety and began weekly high-risk central line rounds in the 3 pediatric units. The pediatric chief quality officer, pediatric quality manager, and a nurse leader rounded on patients who were considered high risk for CLABSI (eg, patients with femoral lines, patients with central lines older than 2 weeks). The purpose of the rounding was to identify barriers to appropriate care and help remove them. An example was a patient who had a documented allergy to chlorhexidine gluconate (CHG), but discussion with the patient's mother revealed that the child had had only a localized reaction. CHG was trialed on a small patch of the child's skin with no irritation, and the patient was able to restart CHG bathing. The team also began using SecurePortIV (Adhezion Biomedical), a sterile glue used to reduce line migration (including PICCs), and the glue is now used for line securement in all pediatric patients. Pediatric CLABSI events declined by 57.1% in the 12 months following the intervention: from 13 events during the 9-month baseline period to 7 events during the 12-month postintervention period (*P*=0.0454). Median pediatric CLABSI events decreased from 2 to zero (Figure 10).

**Table 6.** Pediatric Unit Central Line–Associated Bloodstream Infection (CLABSI) Prevention Stakeholders and Stand-Down Actions

Stakeholder Group	Stand-Down Action	Follow-On Action
Unit leadership team	• Use Children's Hospitals’ Solutions for Patient Safety guidelines and resources • Begin weekly high CLABSI risk rounds • Create distinct central line order sets for newborn, infant, and pediatric ages	• Routinize/shift to supply chain ordering
Nursing	• Begin using special central line securement devices for very young patients • Remove barriers to chlorhexidine gluconate bathing • Introduce use of glue-type line securement	• Expand securement device use
Medical staff	• Use source algorithm for all blood cultures (central vs peripheral vs both)	• Audit medical staff use of source algorithm (conducted by unit-based medical director)
Performance Improvement	• Audit for Kamishibai card and Children's Hospitals’ Solutions for Patient Safety guideline adherence	• Provide audit results to unit staff weekly
Information Services	• Create PICC line clinical pathway	NA
Supply Chain		• Update central line dressing kit • Collaborate with vendor on subclavian insertion process

NA, not applicable; PICC, peripherally inserted central catheter.

### Performance Beyond 6 Months

While performance continued at an improved level for some of the safety stand-downs (*C difficile* infection [Figure 6] and pediatric CLABSI [Figure 10]), challenges were also seen after 6 months, with partial loss of the beneficial effect on event occurrences for hospital CLABSI and inpatient falls, necessitating continued high focus on ongoing PDSA improvement cycles.

## DISCUSSION

We used safety stand-down events to quickly gain control of deteriorating trends in hospital harm event performance. Process measure auditing showed a high degree of stand-down process completion, indicating that implementation of stand-down processes is feasible in a complex hospital environment. The primary mechanism of action underlying the safety stand-down is the intentional management of human factors in high-stress environments. By temporarily compressing the timeline of multidisciplinary problem-solving from months to weeks, stand-downs rapidly align clinical and administrative teams around a single, highly visible risk. While aggregate long-term data trends can mask acute operational volatility, our run chart analyses demonstrate that these short-term interventions can serve as effective course correctors. Our teams’ experience suggests that they can interrupt negative special-cause variation and provide the immediate stability required for long-term, iterative quality improvement tools to succeed. By focusing stakeholder commitment, team attention, and resources, stand-downs seemed to galvanize action and rally resources beyond ongoing improvement activity such as described in publicly available resources to reduce health care–associated infections.^19^

We observed significant decreases in harm events following most safety stand-downs, including *C difficile* infection, inpatient falls, and CLABSI after the second stand-down. We called a second safety stand-down within a year of the first CLABSI stand-down because the first intervention was not detectable as a special-cause variation. During the second CLABSI stand-down, stakeholders observed that the actions taken after the first CLABSI stand-down did not change processes effectively to reduce CLABSI occurrence. Further, the first CLABSI stand-down involved fewer stakeholders with narrower intervention scopes that failed to alter broader clinical outcomes. Consequently, the 2023 stand-down expanded the stakeholder matrix to include Environmental Services, Pharmacy, and Laboratory Services. During the second CLABSI stand-down, Information Services built an electronic best practice advisory for nursing care plans, Pharmacy worked with providers to bundle intravenous medication orders to reduce central line access, Environmental Services focused on room cleaning audits, and Laboratory Services prioritized measures to reduce blood culture contamination. These interventions were thought to engineer process change more reliably than before and with multidepartmental alignment led to a substantive immediate outcome change.

Why the inpatient falls stand-down was effective at reducing the number of falls but did not have a beneficial effect on falls with harm is unclear. One speculation is that personnel improved their ability to detect and report falls with harm over time, but we have no evidence to support this notion. The stand-down provided a targeted short-term mitigation that likely accounts for the decrease in the aggregate annual fall metrics from 354 in 2022 to 275 in 2023. However, because the stand-down did not alter underlying clinical acuity, affect permanent staffing models, or address processes for specific patient populations (eg, anticoagulated patients, patients with advanced osteoporosis), inpatient falls with harm did not decrease, demonstrating the limitations of short-term, attention-based interventions when detached from permanent structural redesign. We recognized that a redesigned intervention specifically addressing inpatient falls with harm would be needed.

The substantive decrease in *C difficile* infections captured in the immediate postintervention months appeared to drive the downward shift in the 5-year aggregate trend (Figure 6), suggesting that short-term operational interventions can successfully alter long-term performance. At the same time, the effect of some of our safety stand-downs is likely to have been somewhat temporary. While we do not have all applicable data extending beyond 6 months, the data we were able to inspect strongly suggest the need for ongoing PDSA type synergistic process improvement and durable interventions that do not depend on temporarily heightened personnel effort and attention. We surmise that the reasons for this observation lie in the loss of institutional and team member memory of process competency caused by personnel turnover, competing priorities, and organizational mini-crises.

The safety stand-down method we used might remind readers of rapid-cycle improvement methodology. Rapid-cycle deliberate practice is characterized by iterative learning processes (often with simulations) designed to up-skill personnel in the face of severe time pressure, such as was encountered in the early days of the COVID-19 pandemic.^20^ Safety stand-downs do not rely on repetitive processes but rather on convening cross-disciplinary stakeholder groups for a short period of time to quickly gain control over a substantive safety condition. During the safety stand-downs conducted at our medical center, we certainly saw the opportunity for follow-on improvement cycles, many of which were acted upon.

Our observations and results should be interpreted with several limitations in mind. First, the stand-downs were conducted at a single institution so the results may not be readily generalizable. Second, the operational and quality structure present at our hospital may not be present in other organizations, thereby limiting their ability to conduct safety stand-downs. For example, our safety stand-downs were enabled by structural assets, such as strong unit dyad relationships between the unit nursing and medical directors, as well as strong executive physician-nursing quality partnerships. Our hospital intentionally requires the nurse manager and medical director of each nursing unit to collaborate systematically in addressing harm events and improving safety processes, patient experience, and discharge delays. Statistical process control methods, including run charts and application of established run chart rules, support inference regarding nonrandom variation but do not establish causality. We do not have data proving that our safety stand-downs caused outcomes to improve. However, a reasonable supposition is that collective increased effort focused on a singular safety issue (such as *C difficile* harm events) resulted in greater awareness and adherence to processes that could prevent harm. For example, we saw awareness increase during shift and midshift huddles as nursing leaders communicated stand-down actions.

Findings should therefore be interpreted as evidence of a temporal association between safety stand-down implementation and subsequent changes in harm event occurrence. Some of the event trends observed after a safety stand-down could possibly have occurred because of other powerful drivers already at work. An example is remote video monitoring, which has been in place at our hospital for several years to alert unit personnel when patients are at risk of falling. We do not believe, however, that all of our hospital stand-down–related improvements (even just those for which we report complete data) were related to confounding factors not associated with stand-down actions.

## CONCLUSION

The results of our hospital-wide and unit safety stand-down initiatives at a 550-bed academic medical center providing tertiary/quaternary care suggest that safety stand-downs can aid in reversing adverse harm trends in hospital-acquired infections and inpatient falls for at least 6 months. This study contributes to the patient safety literature by providing empirical evidence that safety stand-downs can serve as an effective adjunct to ongoing quality improvement efforts when adverse trends threaten patient safety. Unlike prior reports focused on single conditions or short-term process adherence, this analysis demonstrates repeated application of the stand-down method across multiple harm domains and clinical contexts, with consistent short-term impact. These findings support the inclusion of safety stand-downs as a deliberate, time-limited strategy within the broader health care quality improvement toolkit.

## Notes

*This article meets the Accreditation Council for Graduate Medical Education and the American Board of Medical Specialties Maintenance of Certification competencies for Patient Care, Medical Knowledge, Systems-Based Practice, and Practice-Based Learning and Improvement.*
